# Association between Pre-Transplant Serum Malondialdehyde Levels and Survival One Year after Liver Transplantation for Hepatocellular Carcinoma

**DOI:** 10.3390/ijms17040500

**Published:** 2016-04-05

**Authors:** Leonardo Lorente, Sergio T. Rodriguez, Pablo Sanz, Pedro Abreu-González, Dácil Díaz, Antonia M. Moreno, Elisa Borja, María M. Martín, Alejandro Jiménez, Manuel A. Barrera

**Affiliations:** 1 Intensive Care Unit, Hospital Universitario de Canarias, Ofra, s/n, La Laguna, Santa Cruz de Tenerife 38320, Spain; 2 Intensive Care Unit, Hospital Universitario Nuestra Señora Candelaria, Crta Rosario s/n, Santa Cruz Tenerife 38010, Spain; sergiotomasr@hotmail.com (S.T.R.); mar.martinvelasco@gmail.com (M.M.M.); 3 Department of Surgery, Hospital Universitario Nuestra Señora de Candelaria, Crta Rosario s/n, Santa Cruz Tenerife 38010, Spain; sanzpereda@gmail.com (P.S.); mbargom@yahoo.es (M.A.B.); 4 Department of Physiology, Faculty of Medicine, University of the La Laguna, Ofra, s/n, La Laguna, Santa Cruz de Tenerife 38320, Spain; pabreu@ull.es; 5 Department of Digestive, Hospital Universitario Nuestra Señora de Candelaria, Crta Rosario s/n, Santa Cruz Tenerife 38010, Spain; ddiazbet@gmail.com (D.D.); antoniamorenotenerife@gmail.com (A.M.M.); elisaajrob@yahoo.es (E.B.); 6 Research Unit, Hospital Universitario de Canarias, Ofra, s/n, La Laguna, Santa Cruz de Tenerife 38320, Spain; ajimenezsosa@gmail.com

**Keywords:** MDA, hepatocellular carcinoma, liver transplantation, mortality, outcome

## Abstract

Previous studies have found higher levels of serum malondialdehyde (MDA) in hepatocellular carcinoma (HCC) patients compared to healthy controls and higher MDA concentrations in tumoral tissue of HCC patients than in non-tumoral tissue. However, the association between pre-transplant serum levels of MDA and survival in HCC patients after liver transplantation (LT) has not been described, and the aim of the present study was to determine whether such an association exists. In this observational study we measured serum MDA levels in 127 patients before LT. We found higher pre-LT serum MDA levels in 15 non-surviving than in 112 surviving patients one year after LT (*p* = 0.02). Exact binary logistic regression analysis revealed that pre-LT serum levels of MDA over 3.37 nmol/mL were associated with mortality after one year of LT (Odds ratio = 5.38; 95% confidence interval (CI) = from 1.580 to infinite; *p* = 0.007) adjusting for age of the deceased donor. The main finding of our study was that there is an association between serum MDA levels before LT for HCC and 1-year survival after LT.

## 1. Introduction

Hepatocellular carcinoma (HCC) is the most frequent primary malignancy in the liver, one of the most frequent malignancies and the second most frequent cause of cancer-related death in the world. Globally, there are approximately 600,000 new diagnoses of HCC each year and 750,000 deaths due to HCC. Liver transplantation (LT) is generally considered the treatment of choice for selected HCC patients since the primary tumor is removed and liver failure is treated [1,2,3,4,5,6,7,8,9,10].

Oxidative state has been suggested to play a role in the progression of chronic liver disease and in hepatocarcinogenesis. In addition, different antioxidant drugs have been shown to modulate oxidative stress and prevent the appearance of HCC [11,12,13,14].

Oxidative stress leads to peroxidation of membrane lipids, and this process generates a variety of end products, including malondialdehyde (MDA). MDA is a low-molecular weight aldehyde produced by the attack of free radicals to polyunsaturated fatty acids during cellular membrane phospholipid degradation. It is released into extracellular space and finally reaches the bloodstream. Thus, MDA has been used as a lipid oxidation biomarker [15,16].

Previous studies have reported higher serum levels of MDA in HCC patients than in healthy controls [17,18,19], and higher MDA concentrations in tumoral tissue of HCC patients than in non-tumoral tissue [20]. However, the association between pre-transplant serum levels of MDA and survival of HCC patients after liver transplantation (LT) has not been previously reported, and the objective of the present research was to study whether such an association exists.

## 2. Results

**Figure 1:**
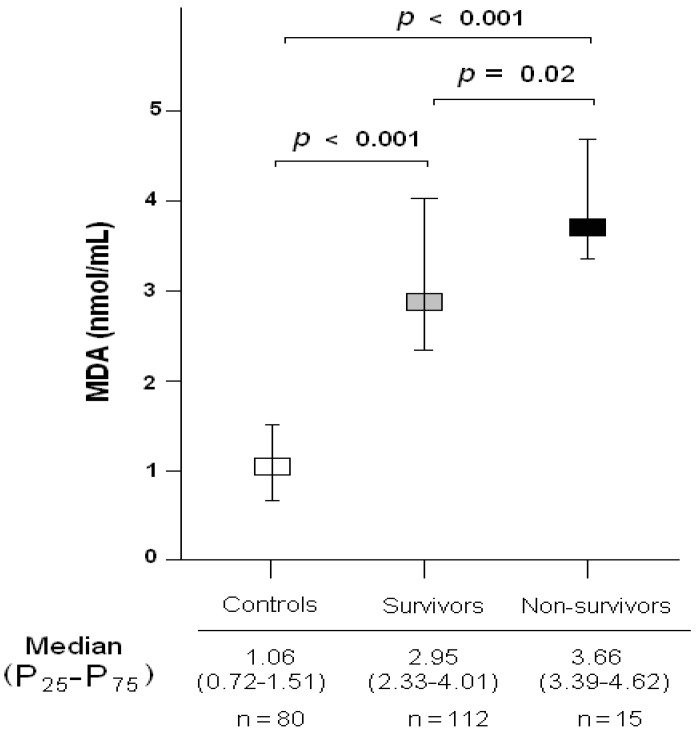
Serum malondialdehyde (MDA) levels in healthy controls, and in 1-year survivors and non-survivors undergoing liver transplantation for hepatocellular carcinoma. P_25_–P_75_ are percentile 25 and 75.

We found higher serum levels of MDA in both surviving and non-surviving patients at 1 year after LT for HCC than in healthy controls (*p* < 0.001, Figure 1). There were no significant differences in age or gender between patients and controls (Table 1).

Table 2 shows demographic and clinical variables of non-surviving (*n* = 15) and surviving patients (*n* = 112) at 1 year after LT. We found no differences between non-survivors and survivors regarding sex, age of LT recipients, ABO blood type, Child-Pugh score, model for end-stage liver disease (MELD) score, Milan criteria, serum alpha-fetoprotein (AFP) levels, portal hypertension, number of nodules, size of nodules, tumor differentiation, infiltration, microvascular invasion, macrovascular invasion, pre-LT treatment or LT technique. However, non-survivors at 1 year after LT had received organs from older deceased donors (*p* = 0.02) and showed higher serum levels of MDA (*p* = 0.02) compared to survivors.

On exact binary logistic regression analysis, serum levels of MDA above 3.37 nmol/mL were associated with mortality after one year of LT (Odds Ratio = 5.38; 95% CI = from 1.580 to infinite; *p* = 0.007) adjusting for deceased donor age (Table 3).

**Figure 2:**
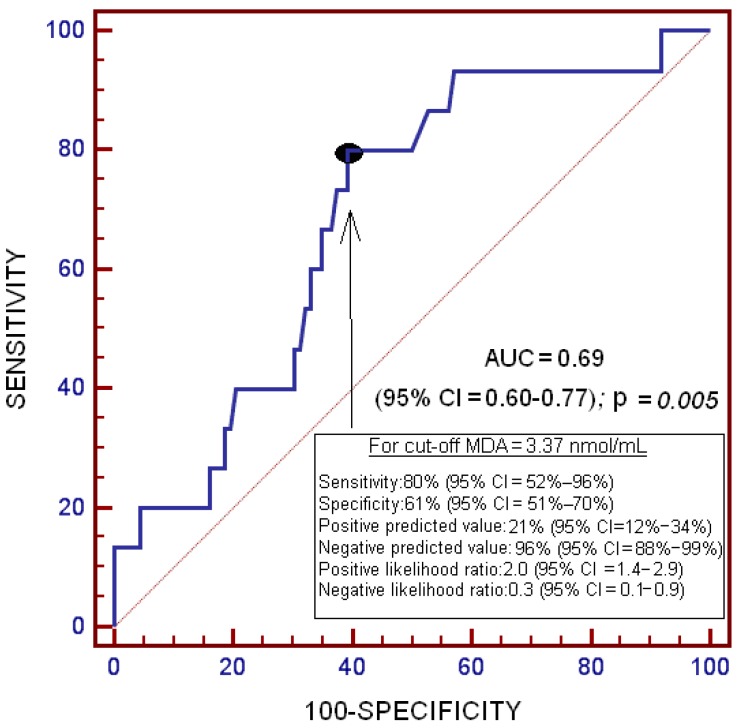
Receiver operator characteristic analysis using serum MDA levels as a predictor of death at 1 year after liver transplantation for hepatocellular carcinoma.

On receiver operator characteristic (ROC) analysis, the area under the curve (AUC) for serum levels of MDA to predict death at 1 year after LT was 0.69 (95% CI = 0.601–0.769; *p* = 0.005) (Figure 2).

**Figure 3:**
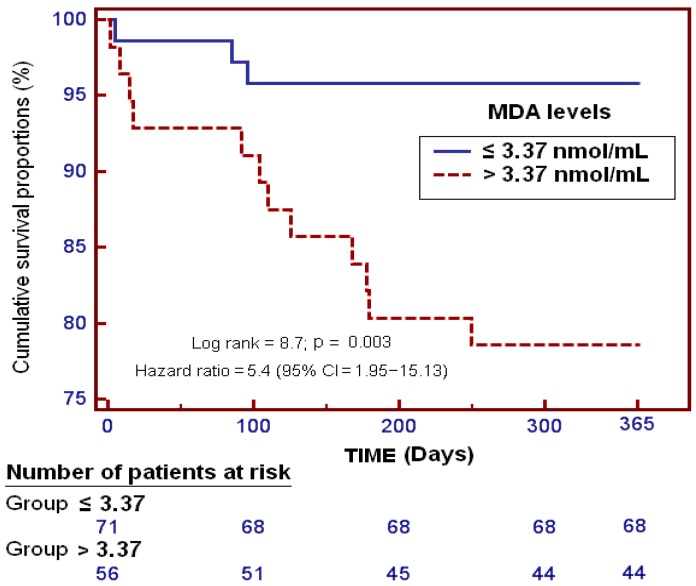
Survival curves at 1 year in patients undergoing liver transplantation for hepatocellular carcinoma using serum MDA levels higher or lower than 3.37 nmol/mL.

Kaplan–Meier survival curves showed that patients with serum levels of MDA above 3.37 nmol/mL had a higher probability of death at 1 year after LT (log-rank = 8.7; Odds Ratio = 5.4 (95% CI = 1.95–15.13); *p* = 0.003) than patients with lower serum levels of MDA (Figure 3).

## 3. Discussion

The most relevant finding of the present study was an association between pre-LT serum levels of MDA and 1-year survival after LT. Pre-transplant serum levels of MDA were higher in non-surviving compared to surviving patients.

We observed higher pre-LT transplant serum levels of MDA in HCC patients than in healthy controls, which is consistent with the findings of previous investigators showing higher serum MDA levels in HCC patients than in healthy controls [17,18,19], in patients with chronic liver disease than in healthy controls [21,22], and the fact that HCC patients present higher free radical intensity in erythrocytes than healthy controls [23]. In addition, higher MDA concentrations have been found in the tumoral tissue of HCC patients than in non-tumoral tissue [20].

One-year survival after LT for HCC patients varies between 79% and 93% [24,25,26,27]; the survival rate in our study (88.2%) fell within this range.

HCC patients with higher serum concentrations of derivatives of reactive oxygen metabolites (d-ROM) present more disease recurrence after curative treatment by radiofrequency ablation or surgical resection [28]. In addition, in patients before LT, non-survivors showed higher circulating lipid peroxide levels than survivors [29]. To our knowledge, the present study is the first to report lower pre-transplant serum MDA levels in survivors than non-survivors at 1 year after LT. In addition, it is the first to report an association between serum MDA levels before LT and 1-year post-transplant survival. These findings are consistent with the results of previous studies that have reported an association between circulating MDA levels and mortality in patients with brain trauma injury [30], brain infarction [31], and sepsis [32,33].

Taken together, our findings indicate that alteration of the oxidative state may be of great pathophysiological significance in HCC patients undergoing LT. Higher circulating levels of MDA in HCC patients than in healthy controls, and in 1-year non-survivors than in surviving patients, represents higher lipid peroxidation due to overproduction of reactive oxygen species (ROS) and reactive nitrogen species (RNS) caused by the imbalance between pro-oxidant and antioxidant systems.

Various factors have been linked with worse outcome in HCC patients undergoing LT, such as tumor size, tumor number, degree of differentiation, hepatic microvascular invasion, hepatic macrovascular invasion, serum alpha-fetoprotein (AFP) levels, outside Milan criteria and infiltration [7,34,35]. In the present study, however, we only found differences in liver donor age, which was lower in surviving patients compared to non-survivors at 1 year after LT.

The development of oxidant state modulators could be a new class of treatment for patients with HCC undergoing LT. The use of different antioxidant agents reduces MDA levels in animal models of sepsis [36,37,38,39] and of trauma brain injury [40,41,42], and also in clinical trials with asphyxiated newborn infants [43], septic newborns [44], adult burn patients [45], acute ischemic stroke patients [46,47,48], and trauma brain injury [49]. In addition, the administration of different antioxidant agents reduces mortality in clinical trials with adult burn patients [45], and trauma brain injury [49]. The potential role of oxidative state in hepatocarcinogenesis, and the use of drugs with antioxidant effects to prevent the development of HCC in patients with chronic liver disease have been suggested [11,12,13,14]. In addition, since non-surviving patients at 1 year showed higher pre-transplant serum MDA levels than surviving patients, the use of drugs with antioxidant effects could be a new treatment to improve the prognosis of those patients, especially those with higher oxidative state.

Our present study has some limitations. First, it was a single-center study and the results may not be extrapolated to patients treated at other institutions. Second, the determination of other compounds of antioxidant and oxidant states could be desirable to better evaluate this balance. Third, other potentially confounding factors not related to sickness (e.g., diet) could have affected MDA serum levels. Fourth, the association that we found between elevated serum MDA levels and reduced survival does not necessarily imply causality, and we have not analyzed the impact of drug modulators of oxidant state; thus, antioxidant drugs may not impact the prognosis.

## 4. Materials and Methods

### 4.1. Design

We performed a retrospective, observational, single-center study with prospective data collection from 127 HCC patients undergoing orthotopic LT from brain death donors in the period between January 1996 to August 2014 at the Hospital Universitario Nuestra Señora de Candelaria (Santa Cruz de Tenerife, Spain). The Institutional Review Board of the Hospital Universitario Nuestra Señora de Candelaria approved the study (permission code PI-33/15; persmission date 30 July 2015). Written informed consent was provided by patients or their family members.

### 4.2. Variables Recorded

The variables recorded for each patient were as follows: sex, age of LT recipient, age of LT donor, AB0 blood type, Child-Pugh score [50], model for end-stage liver disease (MELD) score [51] by hepatic function, Milan criteria [52] before and after LT, serum alpha-fetoprotein (AFP) levels, portal hypertension (assessed clinically or by hepatic venous pressure gradient), number of nodules, size of nodules, tumor differentiation, infiltration, microvascular invasion, macrovascular invasion, pre-LT treatment, LT technique, and serum MDA concentrations.

### 4.3. End-Point

The end-point was survival at 1 year after LT.

### 4.4. Blood Samples and Serum Malondialdehyde Level Analysis

Serum blood samples were collected from 127 patients with HCC before LT (approximately 2 h previous to LT) and from 80 healthy controls. We used the thiobarbituric acid-reactive substance (TBARS) method to determine serum levels of MDA levels, such as was described by Kikugawa *et al.* [53]. The pink complex of samples was extracted in n-butanol. The samples were deposited in 96-well plates and read at 535 nm using a microplate spectrophotometer reader (Benchmark Plus, Bio-Rad, Hercules, CA, USA). Serum concentrations of MDA were expressed in nmol/mL, and the assay detection limit was 0.079 nmol/mL. The coefficient of intra-assay variation was 1.82% and the coefficient of inter-assay variation was 4.01%. All determinations were carried out in the Physiology Department of the Medicine Faculty of La Laguna University (Santa Cruz de Tenerife, Spain) by laboratory technicians blinded to clinical data.

### 4.5. Statistical Methods

Continuous variables are described as medians and interquartile ranges, and categorical variables as frequencies and percentages. We used Mann–Whitney *U* test for comparisons of continuous variables between surviving and non-surviving patients at 1 year after LT, and chi-square test for the comparisons of categorical variables between groups. We plotted a receiver operator characteristic (ROC) curve using serum levels of MDA as the prognostic variable and survival at 1 year after LT as the classification variable. Youden J index was used to select the cut-off prognostic value of serum MDA level. Youden J Index is defined as maximum (sensitivity c + specificity c −1), where c ranges over all possible criterion values [54]. Graphically, Youden J Index is the maximum vertical distance between the ROC curve and the diagonal line. Moreover, using the positive likelihood ratio (sensitivity/(1 − specificity)), the serum MDA level cut-off value is the same (3.37 nmol/mL). Survival analysis was carried out using Kaplan-Meier curves, and comparisons were performed by log-rank test using serum MDA levels lower/higher than 3.37 nmol/mL as the independent variable and survival at 1 year after LT as the dependent variable. Exact binary logistic regression analysis was carried out to determine the independent contribution of serum levels of MDA to predict death at 1 year after LT, controlling for deceased donor age. Since the number of events (death) was low (*n* = 15), the regression analysis only included two predictor variables, and those variables proving significant on bivariate analysis. We calculated odds ratio (OR) and 95% confidence intervals (CI) to measure the clinical impact of the predictor variables. Differences with a *p* value < 0.05 were considered to be statistically significant. All statistical analyses were carried out with SPSS 17.0 (SPSS Inc., Chicago, IL, USA) and MedCal 15.2.1 (Ostend, Belgium).

## 5. Conclusions

The major finding of our study was an association between serum levels of MDA before LT for HCC and 1-year survival after LT. Serum levels of MDA before LT were higher in non-surviving compared to surviving patients.

## Figures and Tables

**Table 1:** Demographic characteristics of healthy controls and patients with hepatocellular carcinoma (HCC) undergoing liver transplantation.

Demographic Characteristics	Healthy Controls (*n* = 80)	HCC Patients (*n* = 127)	*p*-Value
Female gender—*n* (%)	18 (22.5)	20 (15.7)	0.27
Age—median years (P_25_–P_75_)	56 (46–68)	58 (52–62)	0.71
Serum malondialdehyde levels (nmol/mL)—median (P_25_–P_75_)	1.06 (0.72–1.51)	3.11 (2.39–4.17)	<0.001

**Table 2:** Demographic and clinical characteristics of 1-year survivors and non-survivors undergoing liver transplantation for hepatocellular carcinoma.

Demographic and Clinical Characteristics	Survivors at 1 Year (*n* = 112)	Non-Survivors at 1 Year (*n* = 15)	*p*-Value
Female gender—*n* (%)	20 (17.9)	0	0.13
Age (years)—median (P_25_–P_75_)	58 (52–62)	56 (53–62)	0.84
Age of liver donor (years)—median (P_25_–P_75_)	52 (36–63)	62 (49–72)	0.02
ABO blood type—*n* (%)			
A	53 (47.3)	6 (40.0)	0.87
B	9 (8.0)	2 (13.3)	
O	45 (40.2)	6 (40.0)	
AB	5 (4.5)	1 (6.7)	
Child-Pugh score—*n* (%)			
A	54 (48.2)	10 (66.7)	0.41
B	35 (31.3)	3 (20.0)	
C	23 (20.5)	2 (13.3)	
MELD score—median (P_25_–P_75_)	15 (11–18)	15 (15–18)	0.44
Inside Milan criteria previously to LT—*n* (%)	107 (95.5)	14 (93.3)	0.54
Inside Milan criteria after LT—*n* (%)	94 (83.9)	11 (73.3)	0.16
Serum AFP (ng/dL)—median (P_25_–P_75_)	8.0 (4.0–32.0)	12.0 (4.8–164.9)	0.42
Portal hypertension—*n* (%)	78 (69.6)	11 (73.3)	0.99
Multinodular tumor—*n* (%)	34 (30.4)	5 (33.3)	0.77
Nodule size (cm)—median (P_25_–P_75_)	3.0 (2.0–3.5)	3.2 (1.7–4.6)	0.83
Degree of tumor differentiation—*n* (%)			
Well	84 (75.0)	12 (80.0)	0.55
Moderate	25 (22.3)	2 (13.3)	
Poor	3 (2.7)	1 (6.7)	
Infiltration—*n* (%)	36 (32.1)	4 (26.7)	0.77
Microvascular invasion—*n* (%)	24 (21.4)	3 (20.0)	0.99
Macrovascular invasion—*n* (%)	6 (5.4)	0	0.99
Treatment prior to LT—*n* (%)	61 (54.1)	10 (66.7)	0.42
Transplantation technique—*n* (%)			
By-pass	43 (38.4)	6 (40.0)	0.99
Piggy back	69 (61.6)	9 (60.0)	
Serum MDA (nmol/mL)—median (P_25_–P_75_)	2.95 (2.33–4.01)	3.66 (3.39–4.62)	0.02

MELD = model for end-stage liver disease; AFP = alpha-fetoprotein; MDA = malondialdehyde.

**Table 3:** Exact binary logistic regression analysis to predict mortality at 1 year after liver transplantation for hepatocellular carcinoma.

Predictors	Odds Ratio	95% Confidence Interval	*p*-Value
Serum MDA levels > 3.37 nmol/mL	5.38	1.580–infinite	0.007
Age of liver donor (age)	1.04	1.003–infinite	0.04

## Abbreviations

AFP	alpha-fetoprotein
AUC	area under curve
d-ROM	derivatives of reactive oxygen metabolites
HCC	hepatocellular carcinoma
LT	liver transplantation
MDA	malondialdehyde
ROC	receiver operator characteristic
ROS	reactive oxygen species
RNS	reactive nitrogen species
TBARS	thiobarbituric acid-reactive substance
